# Immunoglobulins Content in Colostrum, Transitional and Mature Milk of Bangladeshi Mothers: Influence of Parity and Sociodemographic Characteristics

**DOI:** 10.34763/jmotherandchild.20202403.2032.d-20-00001

**Published:** 2021-01-29

**Authors:** Hashemi Akhter, Farina Aziz, Fahim R. Ullah, Monira Ahsan, Sheikh N. Islam

**Affiliations:** 1 Institute of Nutrition and Food Science, University of Dhaka, Dhaka, Bangladesh; 2 Department of Pharmacy, University of Asia Pacific, Dhaka, Bangladesh; 3 Department of Pharmaceutical Chemistry, University of Dhaka, Dhaka, Bangladesh

**Keywords:** human, immunoglobulins, milk, parity

## Abstract

**Background:**

The study investigated the concentration of IgA, IgM and IgG in colostrum, transitional and mature milk and the effect of parity, age, BMI and family income on secreted immunoglobulin contents of human milk.

**Methods:**

Sequential samples of colostrum, transitional and mature milk were collected from 38 women. Enzymelinked immunosorbent assay was used to analyse the immunoglobulin concentrations.

**Results:**

The study revealed that IgA was the dominant immunoglobulin and mean concentration in colostrum, transitional and mature milk was 5.92 ± 1.50 g/L, 3.85 ± 0.64 g/L and 3.72 ± 0.68 g/L, respectively. Both IgA and IgM levels of colostrum decreased significantly in both transitional (*P* = 0.000) and mature milk (*P* = 0.000), while the concentration of IgG rises significantly in them (colostrum *vs*. transitional milk, *P* = 0.000; and colostrum *vs*. mature milk *P* = 0.011). While maternal age, BMI and family income had no significant influence on the immunoglobulin levels at different stages of lactation, parity showed significant influence on IgG (*P* = 0.03) and IgM (*P* = 0.05) levels of transitional milk and IgA level of colostrum (*P* = 0.05).

**Conclusion:**

The findings suggest that immunoglobulin composition in breast milk is strongly associated with stage of lactation and is likely to be more susceptible to parity than BMI and socioeconomic characteristics.

## Introduction

Maternal milk is considered as the gold standard for infant nutrition because of its influence on the optimal growth and development as well as the neonate immune system. Bioactive factors present in human milk enhance the immature immunologic system of the neonate and support host defence mechanisms against infective and other foreign agents.^1, 2^ As the infant’s gastrointestinal tract and the immune system develop, there is a comparable transition in human milk over time to provide fewer immune factors and more calories and nutrients for growth.^1^

Breast milk contains all the nutrients an infant needs during the first 6 months of life. Breastfeeding further protects against diarrhoea and common childhood illnesses like pneumonia and may also have longer-term health benefits for the mother and child, such as reducing the risk of overweight and obesity in childhood and adolescence. Evidence suggests that initiation of breastfeeding in the first day of life is associated with a significant reduction in the risk of neonatal mortality when compared with delaying breastfeeding for >24 h after birth.^3^ Human colostrum and milk are rich sources of immunologic components that protect suckling infants from infection. Different classes of immunoglobulins present in colostrum and milk confer significant activity against a wide spectrum of harmful micro-organisms. These maternal milk antibodies include IgA, IgG and IgM isotypes as well as the secretory forms of IgA and IgM.^4^ Although all three major classes of immunoglobulins are present in human colostrum and milk, only IgA and IgM are locally produced in the human mammary gland.^5^ These immunoglobulins protect neonates and infants against infection, particularly IgA protects against respiratory tract and gastrointestinal infections.^6^ IgG may play a protective role in the mucosal immune system and may be the most important immunoglobulin in the lower respiratory tract in the newborn period. IgM can also play a role in mucosal defence until serum IgA takes over after the first few months of life. Serum IgA level is then slowly increased, approaching adult levels towards the end of the first year of life. Immunoglobulins of the colostrum and milk protect the neonate until the development of the immune system in the neonate is completed. Thus, the breastfed neonate and infants have lower morbidity and mortality compared with the formula-fed infants.^7^ The antibody-producing cells and the levels of immunoglobulins in colostrum and milk vary at various times after the onset of lactation.^5^ The highest concentrations of IgA and IgM are present in the early colostrum, but the concentration declines sharply 5–6 days postpartum.^8^ Though the concentration of immunoglobulins is lower in mature milk than in colostrum, yet they offer protection until the development of the immune system in the neonate is completed.

Bangladesh has experienced a significant reduction of child mortality including urban–rural disparity over the past few decades, but the mortality among children under the age of 5 is still relatively high (46/1000 live births) despite substantial socioeconomic progress. Malnutrition is one of the major catastrophes in human life, affecting millions of people worldwide. Every year, more than 20 million infants are born weighing <2500 g (WHO defined low birth weight (LBW) as <2500 g)—over 96% of them in low and middle-income countries.^9^ In Bangladesh, newborn infants begin to fight against malnutrition and infection just after their birth mostly due to maternal malnutrition and an unhygienic environment. According to the National Low Birth Weight Survey (NLBWS) of Bangladesh (2003–2004), the percentage of LBW babies in our country is 36%, which is still higher than any other developing country in the subcontinent.^10^ Bangladesh has the highest prevalence of childhood underweight among all countries in the world, except North Korea.^11^ Other risk factors for LBW include low socioeconomic status, low level of parental education, young (<20 years) or old (>35 years) maternal age, primigravida, multigravidity, short stature, lack of antenatal check-ups and iron supplementation during pregnancy, pre-term delivery and lack of adequate rest during pregnancy. LBW is also more common among mothers of parity 1 and >6 than those of parity 1–6, and odds of LBW among primiparity mothers were found to be 5.8 times more likely compared to multipara mothers.^12^

Given the current situation prevailing in the country and acknowledging the importance of breastfeeding, this study assessed the immunoglobulins concentration in colostrum, transitional and mature milk because no information about immunoglobulin levels at different stages of lactation is available as yet in Bangladesh. Attempts were also made to investigate any quantitative difference in levels of immunoglobulins in the different stages of lactation in women of different parity, age, BMI and family income.

## Materials and methods

### Study population and area

The study was conducted with the ethical approval obtained from the Bangladesh Medical Research Council (BMRC), Dhaka. This cross-sectional study was conducted between May and April 2019 at the Dhaka Medical College Hospital (DMCH), one of the major tertiary hospitals in the country. Lactating mothers, aged between 18 years and 40 years, and full-term pregnancies were eligible for inclusion. Participants who had a history of premature or postmature birth, tuberculosis, hypertension, diabetes, cancer or eclampsia were excluded from this study.

### Sample collection

Colostrum, transitional and mature milk (all 1.5 μL) were collected, respectively, on the 2nd day, 2nd week and 4th week of delivery between 10:00 AM and 12:00 AM using a commercial breast pump. Samples were frozen immediately and preserved at −20°C until analysis. A trained research assistant collected samples as well as demographic and anthropometrical characteristics of the mothers under the supervision of a physician (Table 1). Maternal BMI (kg/m^2^) was assessed based on anthropometric criteria (height and weight), referred by WHO expert committee^13^ and maternal characteristics by age, parity and family income.

**Table 1:** Maternal characteristics.

Characteristics	% (*n*)	Mean ± SD
**Maternal age (years)**		22.84 ± 4.8
18–20	34.0 (13)	
21–25	37.0 (14)	
≥26	29.0 (11)	
**Parity**		1.78 ± 0.9
Primiparous	34.0 (13)	
Multiparous	66.0 (25)	
**BMI (kg/m^2^)**		24.65 ± 0.71
<18.5	11.0 (04)	
18.6–25.0	71.0 (27)	
≥25.01	18.0 (07)	
**Family income (BDT )**		6131 ± 287
Up to 4000	29.0 (11)	
4001–8000	47.0 (18)	
≥8001	24.0 (09)	

### Laboratory method

IgG, IgA and IgM concentration in colostrum, transitional and mature milk were estimated by solid-phase indirect enzymelinked immunosorbent assay (ELISA).^14^ About 100 μL of sodium sulphate treated anti-human IgG or IgA or IgM (7.5 μg/mL, Sigma Chemicals, St Louis, MO, USA) coating antigens were pipetted into each well of microtiter ELISA plate (flat bottom, NUNC Immuno plate, Roskilde, Denmark) and incubated overnight at 2–8°C (double plates were coated for each immunoglobulin), washed (×3) with PBS [containing 0.5% Tween 20 (Polyoxyethylene sorbitan monolaurate, Sigma Chemicals, USA)] and dried by gently striking the plate face down on wads of paper towels. The wells were blocked with 100 μL of sheep serum solution (1% v/v in washing buffer), incubated for 1 h at 37°C and treated as afore-described. Then, 100 μL of colostrum and milk preparation (diluted 1:2000 for IgG or 1:60,000 for IgA or 1:1600 for IgM) and serially diluted (12 dilutions: 4 μg/mL, 2 μg/mL, 1 μg/mL, 0.5 μg/mL, and so on) standard immunoglobulins (Sera-pak, Immuno, Bayer, USA) were pipetted into the pre-marked wells and incubated at 37°C for 2 h. After aspirating the excess immunoglobulins, plates were washed and dried similarly. For next stage, around 100 μL of secondary antibody solution [peroxidase-conjugated antihuman IgG or IgA or IGM; Sigma Chemicals; diluted 1:500 with PBS containing 0.05% Tween 20 and bovine serum albumin (0.5 μg/mL)] was pipetted into each well except blank (100 μL of PBS) and incubated and treated as aforementioned. Then ,100 ml of tetramethyl benzidine (TMB; Sigma chemicals, USA) substrate solution [0.1 mg of TMB in 100 μL of dimethylsulphoxide (DMSO) diluted up 10 μL with 0.1 M sodium acetate buffer (pH 6.0), containing hydrogen peroxide] was pipetted into each well and incubated in a dark room at room temperature for 50 min. The enzyme peroxidase reaction was stopped by adding 50 μL of 10% sulphuric acid to each well. Two wells from each duplicate plate were taken for analysis of specific immunoglobulin of every sample (*n* = 4 replica). Finally, reading was taken by ELISA plate reader (Labsystem Multiskan EX, Helsinki, Finland) at 450 nm.

### Statistical analysis

SPSS software (version 20.0, Chicago, IL, USA) was employed for the analysis of the data. Mean differences of immunoglobulins concentration among colostrum and milk were estimated by Student’s *t*-test, and Pearson’s correlation coefficient was used to assess the relationship between lactation stages of the human milk and classes of immunoglobulins. To investigate the influence of maternal characteristics (age, parity and BMI) and family income on the immunoglobulin’s levels (IgA, IgG and IgM) of colostrum and milk, one-way analysis of variance (ANOVA) was performed. Level of significance was set at *P* < 0.05.

## Results

The levels of IgA, IgG and IgM values determined in the colostrum, transitional and mature milk are reported in Table 2.

**Table 2:** Immunoglobulins (IgA, IgG and IgM) concentration (g/L) in colostrum, transitional and mature milk.

Immunoglobulins (*n* = 38)	Concentration (g/L) of immunoglobulins in human milk				Statistics

	Mean ± SD	95% CI (lower-upper)	Ranges	*t*-statistics	Correlation co-efficient
**IgA**					
Colostrum^a^	5.92 ± 1.5	5.45–6.40	3.16–8.97	*t*^ab^ = 7.15 *P*^ab^ = 0.000**	*r*^ab^ = 0.028 *P*^ab^ = 0.94
Transitional milk^b^	3.85 ± 0.64	3.64–4.05	1.35–4.69	*t*^bc^ = 1.02 *P*^bc^ = 0.32	*r*^bc^ = 0.33 *P*^bc^ = 0.04^*^
Mature milk^c^	3.72 ± 0.68	3.50–3.93	1.79–4.43	*t*^ac^ = 8.28 *P*^ac^ = 0.000**	*r*^ac^ = 0.001 *P*^ac^ = 0.543
**IgG**					
Colostrum^a^	0.10 ± 0.03	0.09–0.11	0.04–0.17	*t*^ab^ = =4.03 *P*^ab^ = 0.000^**^	*r*^ab^ = 0.002 *P*^ab^ = 0.537
Transitional milk^b^	0.12 ± 0.02	0.11–0.12	0.08–0.15	*t*^bc^ = 1.00 *P*^bc^ = 0.32	*r*^bc^ = 0.51 *P*^bc^ = 0.001^**^
Mature milk^c^	0.11 ± 0.02	0.11–0.12	0.07–0.17	*t*^ac^ = 2.66 *P*^ac^ = 0.011*	*r*^ac^ = 0.005 *P*^ac^ = 0.654
**IgM**					
Colostrum^a^	0.44 ± 0.10	0.41–0.47	0.23–0.59	*t*^ab^ = 21.37 *P*^ab^ = 0.000^**^	*r*^ab^ = 0.023 *P*^ab^ = 0.345
Transitional milk^b^	0.10 ± 0.02	0.09–0.10	0.04–0.14	*t*^bc^ = 1.28 *P*^bc^ = 0.21	*r*^bc^ = 0.34 *P*^bc^ = 0.04^*^
Mature milk^c^	0.09 ± 0.02	0.08–0.10	0.06–0.15	*t*^ac^ = 20.71 *P*^ac^ = 0.000^**^	*r*^ac^ = 0.005 *P*^ac^ = 0.345

*r* = Pearson’s correlation coefficient ^*^P < 0.05. ^**^P < 0.01. ^a^Colostrum, ^b^Transitional milk, ^c^Mature milk

The correlation of these values with maternal characteristics was reported in four primary categories, i.e. maternal age, parity, maternal BMI and family income (Table 3).

**Table 3:** Association of immunoglobulins concentration at different stages of lactation with maternal characteristics and family income.

Maternal characteristics (*n* = 38)	% (*n*)	Colostrum (g/L)			Transitional milk (g/L)			Mature milk (g/L)		

			Mean ± SD			Mean ± SD			Mean ± SD	

		IgA	IgG	IgM	IgA	IgG	IgM	IgA	IgG	IgM
**Age (years)**										
18–20	34.0 (13)	6.04 ± 1.3	0.10 ± 0.03	0.42 ± 0.1	3.81 ± 0.5	0.13 ± 0.02	0.10 ± 0.02	3.78 ± 0.8	0.12 ± 0.02	0.09 ± 0.02
21–25	37.0 (14)	6.01 ± 1.4	0.10 ± 0.03	0.42 ± 0.1	3.84 ± 0.8	0.12 ± 0.02	0.10 ± 0.02	3.72 ± 0.7	0.11± 0.02	0.09 ± 0.02
≥26	29.0 (11)	5.9 ± 1.5	0.10 ± 0.02	0.41 ± 0.1	3.82 ± 0.6	0.12 ± 0.02	0.10 ± 0.03	3.80 ± 0.7	0.11 ± 0.02	0.09 ± 0.03
***P*-value**		0.98	0.74	0.19	0.78	0.69	0.94	0.99	0.57	0.39
**Parity**										
Primiparous	34.0 (13)	6.58 ± 1.3	0.10 ± 0.022	0.44 ± 0.1	3.73 ± 0.9	0.13 ± 0.02	0.11 ± 0.02	3.78 ± 0.8	0.12 ± 0.02	0.09 ± 0.03
Multiparous	66.0 (25)	5.58 ± 1.5	0.10 ± 0.03	0.44 ± 0.1	3.91 ± 0.5	0.11 ± 0.02	0.09 ± 0.02	3.69 ± 0.6	0.11 ± 0.02	0.09 ± 0.02
***P*-value**		**0.05**	0.56	0.94	0.42	*0.03	**0.05**	0.70	0.76	0.82
**BMI (kg/m^2^)**										
<18.5	11.0 (04)	4.26 ± 1.2	0.08 ± 0.02	0.43 ± 0.1	3.91 ± 0.3	0.11 ± 0.01	0.08 ± 0.02	3.63 ± 0.9	0.10 ± 0.02	0.09 ± 0.00
18.6–25.0	71.0 (27)	6.09 ± 1.3	0.10 ± 0.03	0.44 ± 0.1	3.83 ± 0.7	0.12 ± 0.02	0.10 ± 0.02	3.67 ± 0.7	0.12 ± 0.02	0.09 ± 0.02
≥25.01	18.0 (07)	6.21 ± 1.9	0.10 ± 0.4	0.44 ± 0.1	3.86 ± 0.6	0.12 ± 0.02	0.11 ± 0.02	3.96 ± 0.4	0.11 ± 0.03	0.09 ± 0.03
***P*-value**		0.08	0.69	0.94	0.99	0.37	0.17	0.74	0.47	0.74
**Family Income (BDT)**										
Up to 4000	29.0 (11)	5.06 ± 1.4	0.10 ± 0.03	0.43 ± 0.1	3.88 ± 0.5	0.12 ± 0.02	0.10 ± 0.02	3.72 ± 0.7	0.11 ± 0.02	0.09 ± 0.02
4001–8000	47.0 (18)	5.94 ± 1.5	0.10 ± 0.02	0.44 ± 0.1	3.83 ± 0.8	0.12 ± 0.02	0.10 ± 0.02	3.80 ± 0.7	0.11 ± 0.02	0.09 ± 0.02
≥8001	24.0 (09)	6.22 ± 1.3	0.10 ± 0.04	0.46 ± 0.1	3.85 ± 0.6	0.12 ± 0.02	0.10 ± 0.03	3.56 ± 0.7	0.12 ± 0.03	0.09 ± 0.03
***P*-value**		0.09	0.90	0.73	0.98	0.74	0.92	0.69	0.37	0.96

Results showed that IgA was the predominant immunoglobulin and mean concentration (Table 2) of IgA in colostrum, transitional and mature milk were 5.92 ± 1.50 g/L, 3.85 ± 0.64 g/L and 3.72 ± 0.68 g/L, respectively. The concentration of IgA and IgM showed a sharp fall with the advancement of the time. In contrast, the levels of IgG showed an upward increment in the concentration. Concentration of IgA of colostrum decreased (*P* = 0.000) in transitional milk and mature milk (*P* = 0.000), but the difference between transitional and mature milk was not significant (*P* = 0.32). Significant differences were found in the IgG and IgM level in transitional and mature milk as compared with the colostrum. A significant positive correlation was found in all three immunoglobulin levels in case of transitional and mature milk (IgA: *P* = 0.04, IgM: *P* = 0.04 and IgG: *P* = 0.001) (Table 2).

We analysed the effect of mother’s characteristics (age, parity and BMI) and family income on IgA, IgM and IgG levels at different lactation stages. Maternal age had no significant (all *P* > 0.05) influence on the immunoglobulin levels of colostrum, transitional and mature milk, but parity showed statistically significant influence on IgG (*P* = 0.03) and IgM (*P* = 0.05) level of transitional milk. IgG levels of transitional milk (*P* = 0.03) and mature milk (*P* = 0.76) decreased with increasing parity. Although primiparous mothers had higher IgA concentration in colostrum (6.58 ± 1.30 g/L) than multiparous mothers (5.58 ± 1.50 g/L), yet the difference reached at borderline significant (*P* = 0.05). Moreover, those lactating mothers who had <18.5 BMI (kg/m^2^) had lower IgA in colostrum (4.26±1.2 g/L) than higher BMI (≥25 kg/m^2^; IgA: 6.21±1.9 g/L) or normal BMI (18.6–25 kg/m^2^; IgA: 6.09 ± 1.3 g/L); however, the differences could not reach a significant level (*P* = 0.08). A similar finding was also observed in the case of family income (*P* = 0.09) (Table 3).

## Discussion

The quantitative measurement of the major immunoglobulins in colostrum, transitional and mature milk from the immediate post-partum period through the first 4 weeks of lactation was the first of its kind in Bangladesh. In this study, it was observed that though all three immunoglobulins were present in colostrum, transitional and mature milk throughout the period of lactation, the highest concentrations of IgA and IgM were present only in the early colostrum. The concentration of immunoglobulins declined sharply within 15 days postpartum as the free flow of milk and the overall process of lactation established.

It is evident from the results and findings from similar studies that IgA levels in individual colostrum can vary widely. Mean values of IgA in colostrum recorded range from 5.92 g/L reported in this study to 9 g/L reported in a study conducted on healthy Kiwi mothers.^15^ These means were in agreement with other values in the literature. According to the previous Indian studies,^16, 17^ the mean IgA content of colostrum was found to be 3.35 g/L and mature milk was 1.1 g/L. Mata and Richard^18^ showed that IgA was present in initial colostrum to the extent of 4.1 g/L which decreased to an average 1.8 g/L, 2 weeks after parturition. Goldman et al.^19^ also predicted that IgA decreased during the first 12 weeks but not thereafter. In a survey involving 20 Guatemalan mothers, Stoliar et al.^20^ showed that the mean IgA level was 2.6 g/L in colostrum and 0.7 g/L in transitional milk. Stoliar et al.^20^ also confirmed that in the same postpartum age North American mothers had an identical IgA level. Consistent with the findings of other studies,^8, 15, 17, 18, 21^ there was a rapid decline in the concentrations of the IgA in the milk of our lactating mothers as the lactation period progressed. The IgA content (Table 2) decreased from 5.92 g/L at the colostrum to 3.85 g/L and 3.72 g/L at the transitional and mature stages, respectively. IgA levels were distinctively higher in our study than the values reported in other countries such as India,^16, 17, 21^ Guatemala^18^ and North America.^20^ The higher rate of gastrointestinal infection in our country probably influenced the results. Many experiments have shown that intestinal antigenic exposure can result in homing of lymphoid cells from the Peyer’s patches to the different local IgA production sites, including the mammary glands.^22, 23^

IgM was the second most dominant immunoglobulin, which showed a similar decline pattern like IgA. Significant differences were found between the concentrations of IgM in colostrum, transitional and mature milk (Table 2). The mean total IgM values of 0.44 g/L, 0.10 g/L and 0.09 g/L for the three successive stages were somewhat lower than most previously reported means. In a study conducted on Indian mothers, Narula et al.^16^ reported the IgM concentration in colostrum to be 0.58 g/L, which fell sharply in mature milk (0.24 g/L). In another study conducted in New Zealand, Mickleson and Moriarty^15^ reported a mean IgM level in colostrum to be 1.13 g/L and in mature milk to be 0.05 g/L (42 days postpartum). On the contrary, there are reports of mean IgM levels, which were much lower than recorded in this study.^17, 18^ The decline in the IgM level was in agreement with the study conducted by Ogra and Ogra,^8^ which showed that the highest IgM levels in colostrum were observed in the initial phase of the postpartum period and subsequently, the IgM concentration actively declined in milk during the progression of lactation. Our mean value of IgM is somewhat lower than the Indian value^16^ but higher than the reported value in New Zealand.^15^

The range of IgG level was narrower than that of IgA or IgM (Table 2). IgG content of colostrum was found to be lower than that of values found in Indian^16, 21^ (0.59 g/L) and Kiwi mothers (0.53 g/L),^15^ but higher than the values reported by Mata and Wyatt^18^ in Mayan Indian mothers of Guatemala (0.06 g/L). We found that IgG level increased significantly in transitional (0.12 g/L) and mature (0.11 g/L) milk, which was in agreement with some of the previous studies.^24^ In contrast, there were studies^15, 16^ that recorded a declining pattern of immunoglobulin with the progress of lactation. However, some studies showed no significant changes in the levels of IgG during the subsequent stages of lactation.^8, 17^ This result could be attributed to the presence of microbes in a newborn oral cavity, which was acquired during or immediately after delivery from the mother’s genital tract, from nurses, or the environment, and colonised within a few hours after birth.^25^ This triggers the mother’s immune system by newborn saliva to produce antibodies to treat an acute infection. IgM is the first immunoglobulin produced immediately after exposure to an infectious agent and begins to decrease or disappear and be replaced by IgG, which lasts for life and provides permanent immunity to a person.^26^ This explains the decline of IgM and elevation of IgG during the first month of lactation. This can be further strengthened by the fact that at birth, the neonatal serum contains almost no IgA, a small amount of IgM and adult level of IgG, almost of which is passively transferred maternal IgG. Therefore, the differences in the concentrations of immunologic factors in colostrum and mature milk may direct towards adaptability in the recipient infant. These decrease in immunological indices during subsequent lactation stages may be related to the physiological phenomena that enhance the flow of milk post birth and cause dilution of the breast milk content. The differences in the qualities of colostrum, transitional and mature milk can result from compartmentalisation in the mammary glands during the lactation period, thus supporting the complex physical structure of human milk as earlier proposed.

The study revealed that parity had a significant influence on the immunoglobulin production ability during different stages of lactation. This study showed that the IgA level in the colostrum (*P* = 0.05) and IgM and IgG levels in transitional milk (*P* = 0.05 and *P* = 0.03, respectively) of the multiparous mothers (IgA = 5.58 g/L, IgM = 0.09 g/L, IgG = 0.11 g/L) were lower as compared with primiparous mothers (IgA = 6.58 g/L, IgM = 0.11 g/L, IgG = 0.13 g/L) (Table 3), which was in agreement with the study conducted in the rural Gambia by Prentice et al.^27^ In our opinion, the difficulty of life in Bangladesh with fluctuations in food availability, disease outbreak together with inadequate access to health services and poor utilisation affect the secretory performance of the mammary alveoli as a woman feeds through many different pregnancies, often starting at a much younger age and usually feeding for longer periods.^28^ It is reported that 72.8% of women gave first birth at <20 years of age with a mean age at first birth 18.74 years in our country^29^ as there is social and financial pressure to marry young.^30^ This can be correlated to the significantly higher immunoglobulin level in relatively young primiparous mothers as younger mothers, aged 17–21 years, during their first pregnancy have higher protein concentrations within their breast milk due to more physiologically active secretory cells within the mammary alveoli. Parity in such circumstances may indeed be a major determinant of a women’s immunosecretory ability. Lactation and pregnancy are each very energetically demanding processes. Therefore, the risk of depletion of nutrient stores in the mother may increase, particularly among women with limited access to food. Our results contradicted the previously reported increase of IgA concentration in milk of multiparous women in comparison with primiparous women.^31^ However, another study in India,^16^ reported that there was no significant diference between the mean IgA and IgM of colostrum and milk of primiparous and multiparous mothers. It can be suggested that previous pregnancies and breastfeeding experiences may differently influence the immunoglobulins content of maternal milk according to local and/or environmental conditions.

In Bangladeshi mothers, we were unable to demonstrate any correlation between maternal characteristics such as age, BMI, family income and milk concentration of any three immunoglobulins investigated. The study suggested that these characteristics did not seem to interfere with the production and transfer of immunoglobulins into milk. Our findings were in agreement with the data published in several other studies.^17, 31, 32^ It was noted, however, that mean concentrations of IgA in colostrum examined during this time interval were highest in lactating mothers with higher BMI (≥25 kg/m^2^; 6.21 g/L) and higher family income (≥8000 BDT; 6.22 g/L), but diferences between these values and the means of other BMI and income groups were not statistically significant (Table 3). As low family income can result in limited access to adequate diets in case of poor communities, this finding is of considerable public health significance.^21^

The present study did not record the gestational age in maternal characteristics, which can be considered as a limitation of this work. Gestational age specifies the pregnancy period, and thus overall milk quality. The gap between successive pregnancies was not evaluated in the study, which limits the ability to anticipate whether it was involved in producing a suboptimal outcome for both pregnancy and lactation. Furthermore, smoking and drinking habits among women in Bangladeshi community are unusual, almost non-existent, and none of the participating mothers possessed these habits. It is also possible that if larger sample groups were used, the significance of the differences between the protein concentrations in diferent variable groups might then increase.

## Conclusions

Post-birth physiological phenomena in mothers may be associated with the change in immunoglobulin levels during different stages of lactation. The results of this study indicate that immunoglobulin levels (IgA in colostrum, IG and IgM in transitional milk) were significantly higher in primiparous mothers. This study provides an exciting opportunity to advance our knowledge of parity and sociodemographic-based changes in breast milk. Understanding the changes in human milk composition provides an important tool for the management of infant feeding, particularly of fragile, high-risk infants. This is particularly important in our country where a large majority of children under the age of 5 years sufer from malnutrition. Further research should be undertaken to investigate the relationship between the changes in the immunoglobulin levels and their relationship to newborn’s disease.

## Key points

- Human colostrum and milk are rich sources of immunologic components that protect suckling infants from infection.
- There was a rapid decline in the concentrations of the IgA in the milk of the lactating mothers as the lactation period progressed.
- The levels of IgG in colostrum continued to decrease with the advancement of the lactation period.
- Parity had a significant influence on the immunoglobulin production/secretion ability during different stages of lactation.
- Maternal characteristics, such as age, BMI and family income, do not seem to interfere with the production and transfer of immunoglobulins into milk.
